# Alstroemeria yellow spot virus (AYSV): a new orthotospovirus species within a growing Eurasian clade

**DOI:** 10.1007/s00705-018-4027-z

**Published:** 2018-10-04

**Authors:** A. Hassani-Mehraban, A. M. Dullemans, J. Th. J. Verhoeven, J. W. Roenhorst, D. Peters, R. A. A. van der Vlugt, R. Kormelink

**Affiliations:** 1 Wageningen University and Research, Droevendaalsesteeg 1, 6708 PB Wageningen, The Netherlands; 2 The National Plant Protection Organisation (NPPO) of the Netherlands, P.O. Box 9102, 6700 HC Wageningen, The Netherlands

## Abstract

An orthotospovirus distinct from all other orthotospoviruses was isolated from naturally infected alstroemeria plants. Disease symptoms caused by this virus mainly consisted of yellow spots on the leaves based on which the name alstroemeria yellow spot virus (AYSV) was coined. A host range analysis was performed and a polyclonal antiserum was produced against purified AYSV ribonucleoproteins which only reacted with the homologous antigen and not with any other (established or tentative) orthotospovirus from a selection of American and Asian species. Upon thrips transmission assays the virus was successfully transmitted by a population of *Thrips tabaci*. The entire nucleotide sequence of the M and S RNA segments was elucidated by a conventional cloning and sequencing strategy, and contained 4797 respectively 2734 nucleotides (nt). Simultaneously, a next generation sequencing (NGS) approach (RNAseq) was employed and generated contigs covering the entire viral tripartite RNA genome. In addition to the M and S RNA nucleotide sequences, the L RNA (8865 nt) was obtained. The nucleocapsid (N) gene encoded by the S RNA of this virus consisted of 819 nucleotides with a deduced N protein of 272 amino acids and by comparative sequence alignments to other established orthotospovirus species showed highest homology (69.5% identity) to the N protein of polygonum ringspot virus. The data altogether support the proposal of AYSV as a new orthotospovirus species within a growing clade of orthotospoviruses that seem to share the Middle East basin as a region of origin.

**Electronic supplementary material:**

The online version of this article (10.1007/s00705-018-4027-z) contains supplementary material, which is available to authorized users.

## Introduction

Members of the *Tospoviridae* continuously cause significant losses in both mono- and dicot crops worldwide [1, 2]. Although exact numbers indicating the economic impact of orthotospoviruses are not available, annual yield losses have been estimated to exceed at least 1 billion US$ for the type species *Tomato spotted wilt virus* (TSWV) only [3]. Due to this TSWV ranks high among plant viruses of economic importance worldwide [1, 4–6]. Orthotospoviruses are widespread and transmitted by at least 14 different thrips species [2, 7] in a circulative and propagative manner. Virus particles are membrane-bound with a quasi-spherical morphology of about 80-120 nm in diameter and contain a tripartite ssRNA genome of negative and ambisense polarity [6]. The L RNA is of entire negative polarity and codes for only one gene in the viral complementary (vc) sense, the RNA-dependent RNA polymerase (RdRp; L protein) required for viral replication. The M and S RNA both have an ambisense gene arrangement and contain two ORFs separated by a non-coding intergenic region (IGR). The M RNA encodes a non-structural (NS_M_) protein, in the viral (v) sense, that is involved in cell-to-cell movement and a glycoprotein precursor (G_N_-G_C_) in the vc-sense required for (receptor-mediated) entry into the midgut-epithelium of thrips and vector-specificity [8–13]. The S RNA similarly encodes the non-structural protein (NSs) in the v-sense that acts as suppressor of RNA silencing and supports efficient virus transmission by the vector, and the major structural nucleocapsid protein (N) in vc-sense [14–18]. Although host range, vector specificity and serology of the N protein are often used to identify and/or characterize orthotospoviruses, they only become assigned as a new species if their N protein exhibits less than 90% sequence homology to any of the established orthotospovirus species [2, 19, 20].

Ever since TSWV re-emerged in the 1980’s and started its world wide spread by the introduction and spread of *Frankliniella occidentalis*, new viral species have been discovered. This has led to the identification of a current total of 29 orthotospovirus species of which 11 are officially recognized by the ICTV while the remaining, listed as tentative species [2, 20–26]. During the past two decades many viruses have been observed and identified in ornamental crops of which some cause significant economical losses [27].

Currently, alstroemeria is one of the most important and successful cut flowers and its global production is still increasing [28, 29]. However, viral infections place a large constraint on the production levels and limit global trading [30–32]. To date 18 viruses have been reported from alstroemeria plants (Table S1) [29, 32–42] and among those are isolates from five different orthotospovirus species *i.e.*, TSWV, impatiens necrotic spot virus (INSV), iris yellow spot virus (IYSV) [32], tomato yellow ring virus (TYRV) [35] and alstroemeria necrotic streak virus (ANSV) [34]. From these, TSWV has the largest (natural and experimental) host range, exceeding more than 1350 species. INSV infects at least 300 host species, and mainly ornamentals [7], while IYSV and TYRV have a smaller host range and are frequently found in onion, leek and a few ornamentals [12, 35, 43, 44]. ANSV has been isolated most recently from alstroemeria in Colombia and, together with TSWV and INSV, clusters within the clade of American/New-World orthotospoviruses [2].

**Fig. 1:**
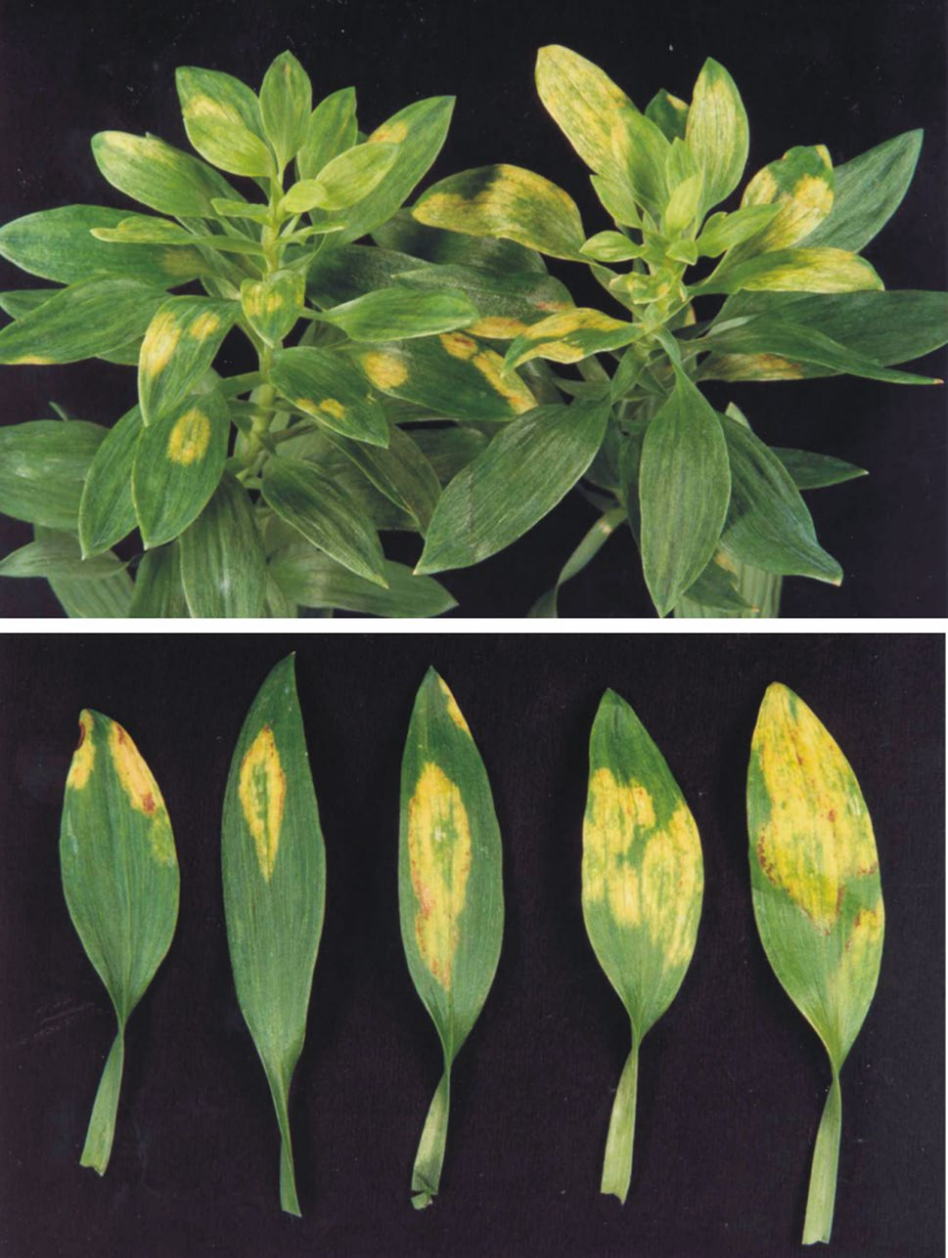
Symptoms of alstromeria yellow spot virus (AYSV). Yellow spots on alstroemeria leaves of naturally infected plant

In 2000 an alstroemeria plant showing only chlorotic and yellow spots on the leaves (Figure 1), and reminiscent of a TSWV infection, was received by the Netherlands Inspection Service for Horticulture. Preliminary serological tests with available antisera to a wide range of different orthotospoviruses were negative and indicated the possible emergence of a new orthotospovirus species. Here data is presented on the biological, serological and molecular characterization of this Als-2000 virus isolate to support the proposal of a new tentative orthotospovirus species for which the name alstroemeria yellow spot virus (acronym AYSV) is proposed. The nucleotide sequence of the entire genome was elucidated using a simultaneous conventional and NGS cloning/sequencing approach.

## Materials and methods

### Virus source, virus isolates and host range

Leaf material of suspected virus-infected (Als-2000) alstroemeria was homogenized in PBS buffer (pH 7.0) with 0.01% sodium sulphite and mechanically inoculated to *Nicotiana benthamiana*. Orthotospovirus isolates TSWV BR-01 [19], tomato chlorotic spot virus (TCSV) BR-03 [19], groundnut ring spot virus (GRSV) SA-05 [19], IYSV-NL [45] and TYRV-t [46] used in serological studies, were also maintained on *N. benthamiana*. For a host range analysis, sap from Als-2000-infected alstroemeria leaf material was mechanically inoculated on 29 plant species (at least 2 plants/species) and monitored during 3-4 weeks for symptom expression.

### Ribonucleocapsid purification, SDS-PAGE and serological analysis

Ribonucleocapsid protein (RNP) was purified from Als-2000-infected *N. benthamiana* plants [47] and subjected to 25-45% CsSO_4_ gradient centrifugation. RNP fractions collected were used to immunize rabbits for the production of a polyclonal antiserum. Prior to this, RNP fractions were subjected to a quality/purity check, in comparison to INSV and TSWV RNP preparations, on a 12% SDS-PAGE after denaturation in Laemmli buffer [48]. Rabbits were immunized 2-3 times with 50-100 μg of purified RNP emulsified with incomplete Freund´s adjuvant (1:1, w/v), during intervals of two weeks. Serum was collected two weeks after the last injection and used for the purification of IgG and an the preparation of an IgG-alkaline-phosphatase conjugate. For orthotospovirus differentiation double-antibody sandwich–enzyme-linked immunosorbent assays (DAS-ELISA) were performed using a panel of polyclonal antisera raised against Als-2000, TSWV, TCSV, GRSV, IYSV and TYRV [45–47]. Sap of infected and healthy *N. benthamiana* plants was diluted 1:30 and used as the antigen source and negative control, respectively.

### RNA purification and RT-PCR

Viral RNA was purified either from purified RNPs using 1% SDS and hot phenol extraction followed by ethanol precipitation [9] or from infected plant material using Trizol buffer according to the manufacturer’s instructions (Invitrogen).

In a first approach to obtain Als-2000 viral sequences only a single primer “Asian Termini” (AT; 5΄-CCCGGATCC*AGAGCAATCGAG*-3΄, containing a *Bam*HI restriction site (underlined) and matching the 3’ terminal sequences of Asian orthotospoviruses (italics), was used for first-strand cDNA synthesis using M-MLV Reverse Transcriptase (Promega), and a subsequent PCR amplification [46] using Phusion High-Fidelity DNA Polymerase (New England BioLabs). Since the sequence of the amplicon obtained using the AT primer showed highest homology with the NS_M_ gene of IYSV, a second approach was applied in which Als-2000 sequences were additionally RT-PCR amplified using primers IY1 (5′-CCCGAGGATCC*ATGGCTACCGTTAGGG*-3′) and IY2 (5′-CCCGAGGATCC*AAATTAATTATATCTATCTTTCTTGG*-3′) corresponding to IYSV N gene sequences (in italics) combined with the universal hairpin primer (UHP) (5’-CACTGGATCC*TTTTGTTTTTGTTTTTTG*-3’) as described before [45, 46]. Based on newly obtained sequences for Als-2000, specific primers were designed to verify/obtain additional sequences to complete the entire S RNA nucleotide sequence. Primers Als2000-NS_M_-R (5’-GCTTGGTTTTCTTTCTTTTTCCTTC-3’) and Als2000-G-R (5’-CCTCCTAAAACATATGACTTTCC-3’) were used to amplify the 5’ ends of the NS_M_ respectively G_N_/G_C_ glycoprotein precursor ORFs in combination with the AT termini primer. In analogy to the approach for the S RNA segment, new and specific primers were designed to verify and/or obtain sequences for the intergenic region of the M RNA segment. All PCR fragments obtained were cloned into pJET blunt1.2 vector (Fermentas) and their sequences determined (Eurofins).

### Sequence analysis of S and M RNA segments

Contigs for the M and S RNA segments were assembled from obtained sequences using DNAStar software and used to search the GenBank database using BLASTn and BLASTp. Multiple sequence alignment and phylogenetic analysis were performed using MEGA 7 software [49]. The G_N_-G_C_ sequence was analyzed for the presence of N- and O-linked glycosylation sites by prediction using the NetNGlyc 1.0 and NetOGlyc 3.1 Servers (http://www.cbs.dtu.dk/services/NetNGlyc/; http://www.cbs.dtu.dk/services/NetOGlyc-3.1/). Potential signal cleavage sites within the glycoprotein were predicted using the SignalP 4.1 server (http://www.cbs.dtu.dk/services/SignalP/). Transmembrane regions within the G_N_-G_C_ precursor protein were predicted using the TMHMM Server v.2.0 program (http://www.cbs.dtu.dk/services/TMHMM/).

### Sequences analysis of Als-2000 by Next Generation Sequencing

For the sequencing of the entire Als-2000 genome using next generation sequencing (NGS) technology, total RNA was isolated from systemically infected *N. benthamiana* leaves with the Qiagen RNeasy plant mini kit according the manufacturers’ procedures (Qiagen). Samples were prepared for HighSeq sequencing according to Verbeek *et al.* [50]. Sequence data were analyzed using the CLC Genomics Workbench 8.5.1 (Qiagen) as earlier described [51]. To obtain and verify the full length sequence of Als-2000 the 5’- and 3’-ends of the viral RNA segments and regions from which only few reads were obtained, were RT-PCR amplified with specific primers and directly sequenced by Sanger sequencing (Macrogen).

### Thrips transmission

To determine the potential of *Thrips tabaci* for transmission of Als-2000, 10 first and early second instar larvae of *T. tabaci* were given an acquisition period of 24h on Als-2000 infected *Emilia sonchifolia* and next transferred to healthy *E. sonchifolia* plants. Plants were kept under cages at room temperature and monitored for symptom expression for two weeks. The presence of Als-2000 in plants was tested by RT-PCR using primers IY1 and IY2. Fragments of expected sizes were collected and the AYSV sequence confirmed by sequence analysis.

## Results

### Host range analysis

From 31 plant species mechanically inoculated with Als-2000, from here onwards referred to as alstroemeria yellow spot virus (AYSV), 17 species did not show any sign of infection, 9 species only showed local symptoms while 5 species displayed local and systemic symptoms (Table 1). Back-inoculation of the isolate from *N. benthamiana* on the alstroemeria cultivar Dimention did not induce any symptoms. The data indicated that the virus had a relatively narrow host range and among the plants tested only dicots showed local or systemic symptoms.

**Table 1:** Host range study of AYSV (Als-2000 isolate) via mechanical inoculation

Plant family and species	Symptom	
	Local	Systemic
***Alstroemeriaceae***		
*Alstroemeria sp.* cv. Dimension	-	-
***Amaranthaceae***		
*Gomphrena globosa*	-	-
***Amaryllidaceae***		
*Allium porrum*	-	-
*Hippeastrum sp*. cv. Orange Souvereigh	-	-
*Hippeastrum sp.* cv. Red Lion	-	-
***Asteraceae***		
*Emilia sonchifolia*	CS, NS	CS, NS
*Zinnia elegance*	-	-
***Balsaminaceae***		
*Impatiens sp.*	NL,NR,N	-
***Chenopodiaceae***		
*Chenopodium amaranticolor*	-	-
*Chenopodium quinoa*	-	-
***Cucurbitaceae***		
*Cucumis sativus* cv. Hokus	-	-
*Cucurbita pepo* cv. Black Beauty	-	-
***Fabaceae***		
*Arachis hypogea*	-	-
*Vicia faba* cv. Wikiem Majort	NL	-
*Vigna unguiculata* cv. Black Eye	NL	-
*Vigna unguiculata* cv. Early Red	CL,NL	S
*Phaseolus vulgaris* cv. Pinto	NL	-
***Iridaceae***		
*Iris hollandicum* cv. Blue Magic	-	-
***Solanaceae***		
*Capsicum annuum* cv. Westlandse Grote Zoete	-	-
*Datura metel*	NL	-
*Datura stramonium*	NL	-
*Physalis floridana*	-	-
*Pisum sativum*	-	-
*Nicotiana benthamiana*	CL	CS, NS, C,RG
*Nicotiana clevelandii*	C,N	C,N,RG
*Nicotiana glutinosa*	NL	-
*Nicotiana hesperis-67A*	CL,NL	NL
*Nicotiana occidentalis-P1*	NL	-
*Nicotiana rustica*	-	-
*Nicotiana tabacum* cv. White Burley	NL,NR	-
*Solanum lycopersicum* cv. Money Maker	-	-

*C* chlorosis; *LC* leaf curling; *CL* Chlorotic lesions; *CS* Chlorotic spot; *N* necrosis; NL necrotic lesions; *NR necrotic rings; NS* necrotic spots; *RG* retardation in growth; *S* symptomless infection

### N protein electrophoresis and DAS-ELISA

**Fig. 2:**
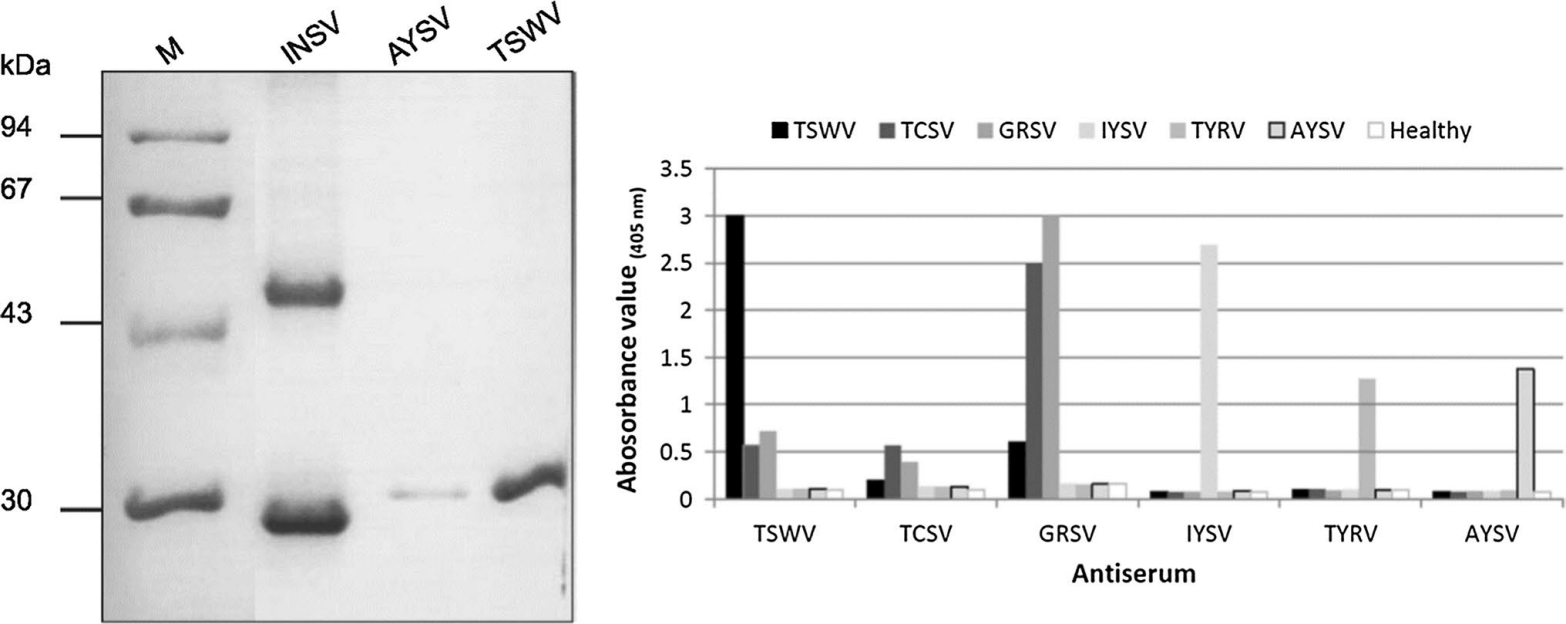
Analysis of the major structural N protein from purified AYSV RNPs and serology. Purified AYSV RNPs were comparatively analyzed to those of TSWV and INSV on SDS-PAGE (left panel). Proteins were stained with Coomassie Brilliant Blue. Size marker is indicated at the left side of the gel. Serological differentiation between AYSV and five orthotospoviruses in DAS-ELISA (right panel) was performed using polyclonal antisera raised against respective N proteins and the extracts from infected plants as antigen source

To comparatively analyze AYSV and serologically differentiate this virus from other orthotospoviruses, AYSV RNPs were purified and analyzed by SDS-PAGE. A major band of approximate M_W_ 30 kDa migrated at a position in between the INSV and TSWV N proteins and likely represented the AYSV N protein (Figure 2, left panel). Polyclonal antibodies produced against the AYSV RNPs were used to analyze its serological relationship to a set of orthotospoviruses. In DAS-ELISA, only leaf extracts of AYSV-infected plants reacted positively with polyclonal antiserum against AYSV, while no positive reaction was observed with antisera raised against other orthotospovirus species (Figure 2, right panel).

### Sequence analysis and characterization of the S RNA, M RNA and L RNA

The entire sequence of AYSV S RNA consisted of 2734 nucleotides (nt) with 5′ terminal (72 nt) and 3′ terminal (71 nt) untranslated regions (UTR) that contain the highly conserved 8 terminal nucleotides present in all orthotospovirus RNA genome segments. AYSV terminal sequences show almost entire base pair complementarity of the first 18 nt with only 3 base pair mismatches (5’-AGAGCAAUCGNNGNAUAA-3’). This complementarity continues, although to a lesser extent, to ~ 60 nucleotides of the UTR sequences and assists in the panhandle formation of the S RNA segment.

The TYRV S RNA contains a vORF starting with an AUG at nucleotide position 73 and terminating with a UAG codon at position 1404, coding for 443 residues of the NS_S_ protein with a predicted molecular mass of 50.1 kDa. The vcORF starts with an AUG at nucleotide position 2663 and terminates with a UAA stop codon at nucleotide position 1845, coding for 272 residues of the N protein with a predicted molecular mass of 30.1 kDa. A noncoding intergenic region (IGR) encompassess nucleotide position 1405 to 1844 and is highly rich in stretches of A and U residues, enabling the putative formation of a hairpin structure (data not shown).

**Fig. 3:**
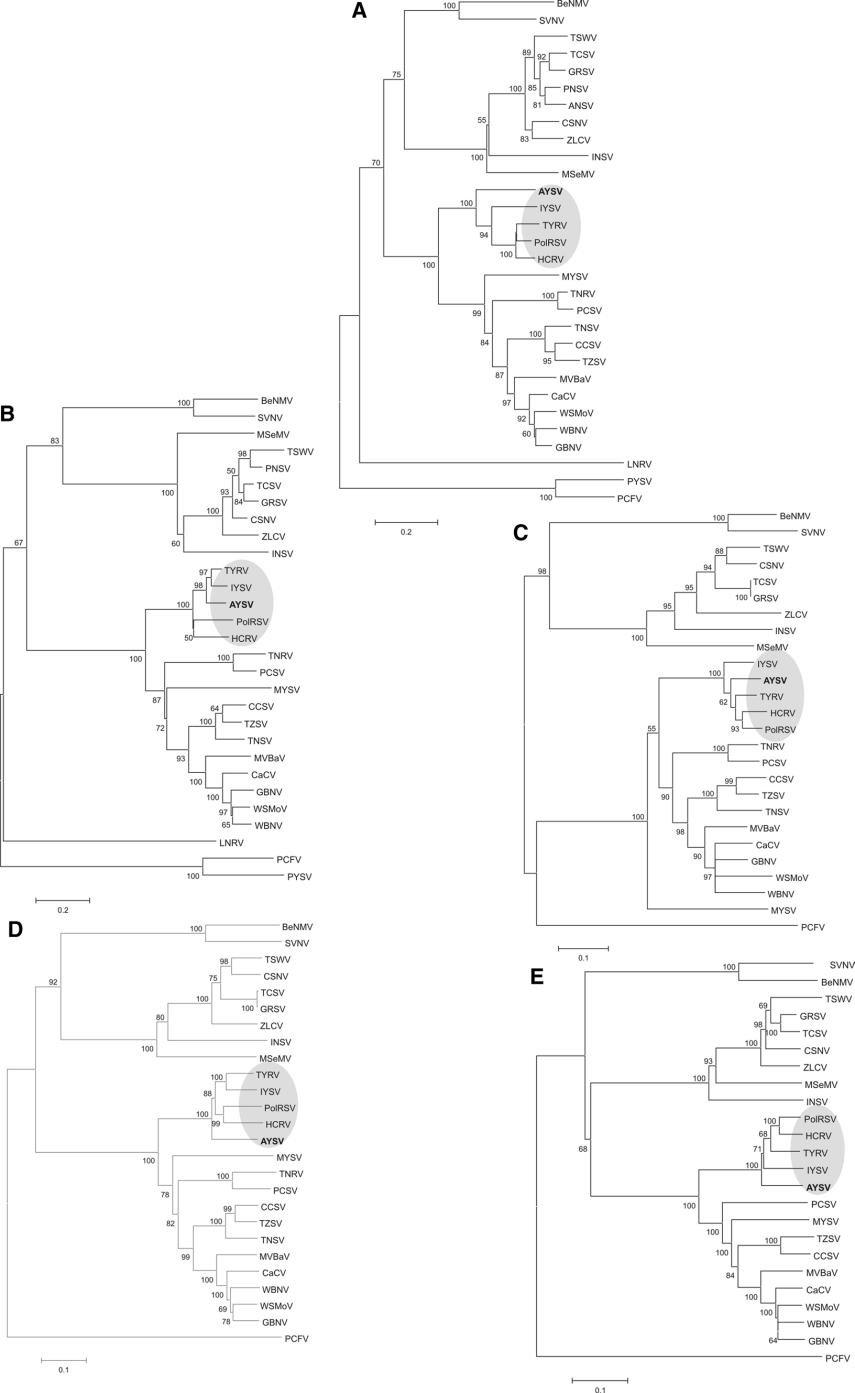
Phylogenetic relationships of alstroemeria yellow spot virus (AYSV) proteins and those of the other tentative and established orthotospovirus species. The trees were inferred based on the alignments of amino acid sequences of the nucleocapsid (N) proteins (A), RNA silencing suppressor (NS_S_) proteins (B), cell-to-cell movement (NS_M_) proteins (C), precursor Glyco (G_N_-G_C_) proteins (D) and RdRp (L) proteins (E) from alstroemeria necrotic streak virus (ANSV), capsicum chlorosis virus (CaCV), calla lily chlorotic spot virus (CCSV), chrysanthemum stem necrosis virus (CSNV), groundnut bud necrosis virus (GBNV), groundnut ringspot virus (GRSV), impatiens necrotic spot virus (INSV), iris yellow spot virus (IYSV), lisianthus necrotic ringspot virus (LNRV), melon severe mosaic virus (MeSMV), melon yellow spot virus (MYSV), peanut chlorotic fan-spot virus (PCFV), peanut yellow spot virus (PYSV), polygonum ringspot virus (PolRSV), tomato chlorotic spot virus (TCSV), tomato necrotic spot virus (TNSV), tomato spotted wilt virus (TSWV), tomato yellow ring virus (TYRV), tomato zonate spot virus (TZSV), watermelon bud necrosis virus (WBNV), watermelon silver mottle virus (WSMoV) and zucchini lethal chlorosis virus (ZLCV) and AYSV (this report). The trees were constructed using the neighbour-joining method implemented in MEGA7.0. Bootstrap values are shown as percentages derived from 1,000 replicates. Those values less than 50% are not shown. GenBank accession numbers of the viral genome sequences from which the viral protein sequences were taken are listed in table S2

Upon multiple sequence alignment of the AYSV N protein to other orthotospoviral N proteins, AYSV N showed highest identity (69.5 %) with polygonum ringspot virus (PolRSV). This homology is well below the threshold for demarcation of new orthotospovirus species and indicated that AYSV tentatively presents a new distinct species. In a phylogenetic analysis based on the N protein, AYSV clusters together with members initially found in Europe and Asia that belong to the so-called Eurasian clade. A similar phylogenetic analysis using AYSV NS_S_ revealed 88.3 % identity to that of TYRV, and supports the relationship of AYSV to the Eurasian clade (Figure 3).

The complete nucleotide sequence of AYSV M RNA consists of 4797 nt, and contains a 5’- (62 nt) and 3’-UTR (51 nt) that is able to form a panhandle structure due to sequence complementarity. The UTRs show almost entire sequence complementarity for 29 nt with only 3 mismatches (5’-AGAGCAAUCGGUGCAANAAUCAAAUNUNU-3’). The vORF starts with an AUG at nucleotide position 63 and terminates with an UAA codon at position 989, coding for the NS_M_ protein with a predicted molecular mass of 34.2 kDa. The AYSV NS_M_ protein shows conserved motifs that belong to the 30K movement protein superfamily [52, 53], including D_155_, G_211_ and P/D-L-X (amino acid residues D_102_S_103_L_104_), and motifs for a phospholipase A2 catalytic site and PLA2 (amino acid residues CMQLNLTS_[197-204]_).

The vcORF codes for the glycoprotein precursor and starts with an AUG codon at nucleotide position 4746 and terminates with UGA at nucleotide position 1300. The M RNA encoding v- and vc-ORFs flank an IGR of 310 nt that runs from nucleotide position 990 to 1299 and, like the S RNA, consists of nucleotide stretches highly rich in A- and U residues. Folding predictions of the IGR sequence reveals the formation of a stable hairpin structure (data not shown).

Using BLASTp, the highest identity of AYSV NS_M_ was found with TYRV NS_M_ (88.7%) whereas its glycoprotein precursor (GP) showed highest identity with that of TYRV GP (81.0%). Phylogenetic analysis based on the NS_M_ protein and the glycoprotein precursor support a classification of AYSV within the Eurasian clade (Figure 3).

**Fig. 4:**
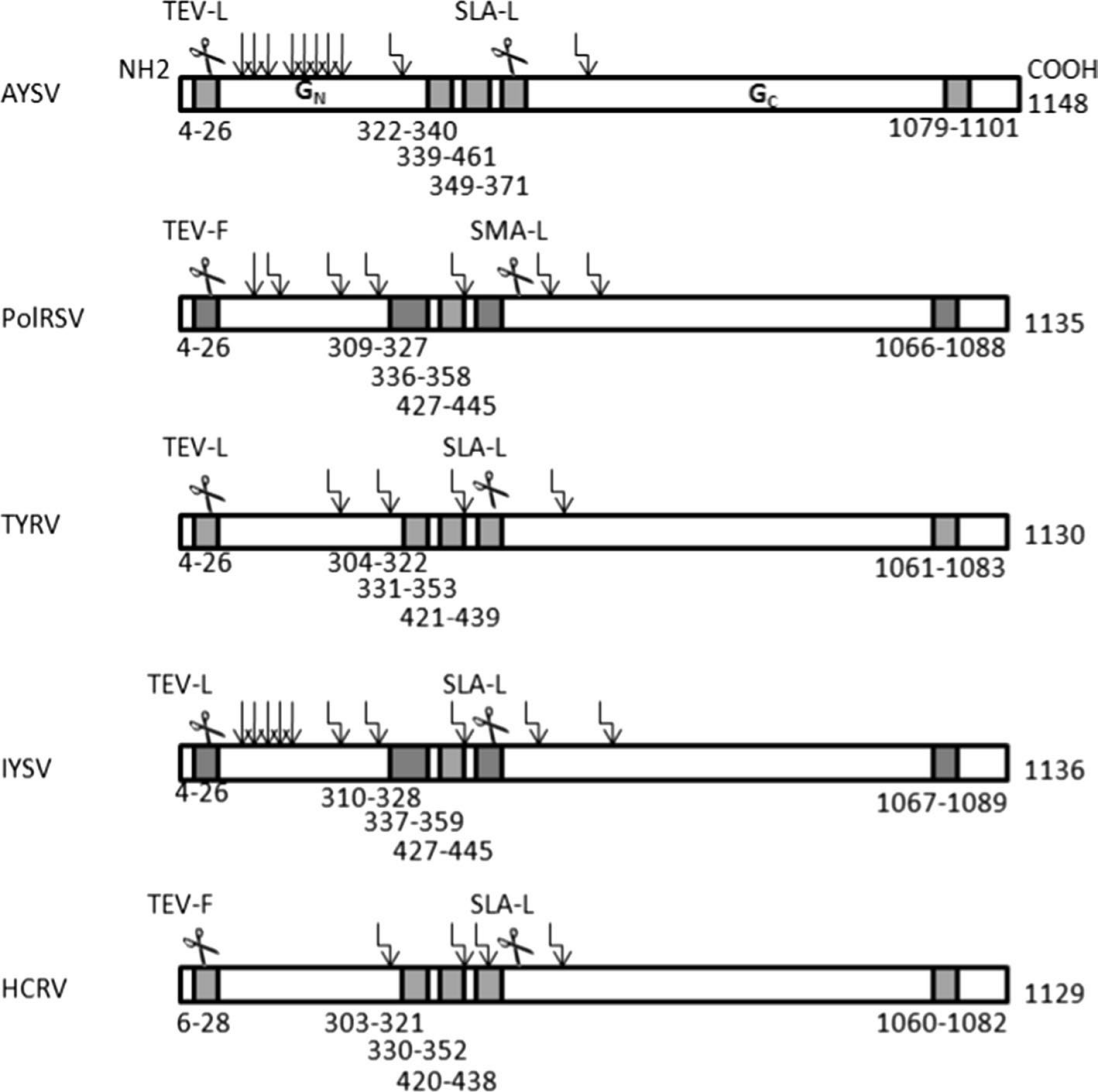
Topology of the glycoprotein precursor of AYSV in comparison to the ones from other Euroasian orthotospoviruses. Predicted glycosylation sites are indicated with arrows op top. Hydrophobic domains representing signal sequences and transmembrane domains are indicated in grey, and their locations within the precursor with the position of amino acids below. Predicted cleavage sites for maturation of the precursor into the mature G_N_ and G_C_ glycoproteins are indicated with scissors with the amino acids sequence of the cleavage sites above it (TEV-L and SLA-L, respectively)

The surface glycoprotein of AYSV was analyzed and compared to those of other Eurasian orthotospovirus species for the presence of N-, O- Glycosylation, peptide cleavage sites and transmembrane domains (Figure 4). Two putative N-glycosylation sites, Asn_319_ and Asn_575_ and eight O-glycosylation sites i.e. Thr_80_, Thr_82_, Thr_84_, Thr_99_, Thr_100_, Thr_101_, Thr_102_ and Thr_105_ were predicted. Two signal peptide sequences with predicted cleavage sites were found at amino acid positions Leu_23_ and Leu_462_. Five (hydrophobic) transmembrane domains were found in the G_N_-G_C_ precursor. Both of the predicted cleavage sites located within the first and fourth transmembrane domains, whereas none of the predicted glycosylation sites mapped within transmembrane domains (Figure 4). A comparison to the G_N_-G_C_ precursor of other Eurasian orthotospovirus species revealed that the AYSV precursor contains the lowest number of N-glycosylation sites and the highest number of O-glycosylation sites. Based on the predicted cleavage sites, the AYSV G_N_-G_C_ precursor is processed into the mature G_N_ and G_C_ glycoproteins of 50.3 and 77.4 kDa, respectively (Figure 4). The AYSV G_N_-G_C_ precursor does not contain a Arg-Gly-Asp (RGD) sequence, a motif earlier found in the TSWV precursor and putatively involved in virus cell attachment [9].

The entire sequence of AYSV L RNA consists of 8865 nt with a 5′ terminal (214 nt) and 3′ terminal (32 nt) UTR that contain the highly conserved 8 terminal nucleotides present in all orthotospoviral RNA segments. AYSV terminal sequences show almost entire base pair complementarity for the first 16 nt with only one base pair mismatch (5’-AGAGCAAUCNAGCAAC-3’).

The L RNA contains a single ORF of 8619 nt in the vc-strand and coding for the putative viral RNA-dependent RNA polymerase (RdRp, L protein) of 2873 aa with a predicted molecular mass of 316.0 kDa. The vcORF starts with an AUG at nucleotide position 8833 and terminates with an UAA stop codon at nucleotide position 212. The L gene of AYSV shares highest aa (87.3 %) identity with the one from TYRV. Analysis of the AYSV L protein revealed the presence of all conserved motifs earlier reported and indicative for the RdRp [54, 55]. The highly conserved region, shared among all characterized orthotospoviral L proteins, is found at aa position 1,275–1,559 of the AYSV L protein. This region contains the essential motifs of viral RdRp proteins from negative strand RNA viruses and includes motif A (aa 1,365–1,378), motif B (aa 1,452–1,471), motif C (aa 1,490–1,499), motif D (aa 1,534–1,544), motif E (aa 1,547–1,559), and motif F (aa 1,275–1,301) [54, 55]. The AYSV RdRp motif sequences are 100% identical to those of TYRV. Phylogenetic analysis of the RdRp shows a clustering of AYSV with TYRV, PolRSV, hippeastrum chlorotic ringspot virus (HCRV) and IYSV (Figure 3).

A summary and comparative analysis of the AYSV L, M and S RNA sequence to those of other Eurasian species is shown in Table 2. While the L RNA sequence has not been elucidated for all Eurasian species, the structural features of the M and S RNA of AYSV show highest similarity to those from PolRSV, the closest related species, with the exception of a differently sized IGR within the S segment (AYSV-IGR_478_ vs. PolRSV-IGR_183_).

**Table 2:** Comparison of structural features of the L, M and S RNA segments of AYSV to those of other Eurasian orthotospoviruses

Virus acronym	AYSV	PolRSV	TYRV	IYSV	HCRV
S RNA*	2,734	2,484	3,061	3,105	2,744
5’ UTR*	72	72	71	70	73
NS_S_**	443	443	443	443	445
IGR*	440	183	762	811	437
N**	272	274	274	273	274
3’ UTR*	71	72	71	70	71
M RNA*	4,797	4,710	4,786	4,838	4,741
5’ UTR*	62	62	62	63	47
NS_M_**	308	308	308	311	308
IGR*	310	263	354	379	330
G_N_-G_C_**	1,148	1,135	1,130	1,136	1,129
3’ UTR*	51	50	50	49	47
L RNA	8865	8893	8877	8880	8908
5’ UTR*	211	230	223	225	253
RdRp**	2,873	2,876	2,873	2,873	2873
3’ UTR*	32	32	32	33	33

* Number of nucleotide residue; ** Number of amino acid residue

The complete L, M and S RNA sequences of AYSV are available under GenBank accession no. MF469033, MF469034 and MF469035 respectively

### Vector transmission

Healthy *E. sonchifolia* plants exposed to *T. tabaci*, that in their larval stages were given access to AYSV-infected plants, revealed chlorotic spots similar to those observed earlier during the host range analyses (Table 1). The presence of AYSV was confirmed and successful transmission by viruliferous thrips was demonstrated by RT-PCR amplification of (partial) AYSV N gene sequences from infected leaf material and subsequent sequence analysis.

## Discussion

Here a tentative new orthotospovirus species has been identified and characterized from alstroemeria, and for which the name alstroemeria yellow spot virus (AYSV) has been proposed. The virus causes yellow spots on alstroemeria, has a narrow experimental host range limited to a few dicots and is transmitted by the vector *T. tabaci*. A polyclonal antiserum has been raised against AYSV RNPs and only reacts positively with the homologous virus and not with any other established orthotospovirus species tested. The nucleotide sequence of the genomic L, M and S RNA segments has been elucidated (8619, 4797 and 2734 nt, respectively) and contains 5 ORFs coding for six mature proteins i.e., the L protein (viral RdRp, 316 kDa), the cell-to-cell movement protein (NS_M_, 34.2 kDa), the precursor (127,7 kDa) to the glycoproteins G_N_ and G_C_, the suppressor of RNA silencing (NS_S_, 50.1 kDa) and the nucleocapsid protein (N, 30.1 kDa). The AYSV sequence shows all structural features typical for orthotospovirus RNA genomes with highest homology between its proteins and those from TYRV. Phylogenetic analysis indicates that AYSV, together with PolRSV, TYRV, HCRV and IYSV, belongs to a growing clade of orthotospoviruses for which the geographical origin seems to be based in the Middle-East [46]. Although the origin of the virus-infected alstroemeria plant could not be indicated with certainty, it was provided by a grower that at that time had simultaneously received shipments of alstroemeria plants from Colombia and Iran. Considering the relation of AYSV to the orthotospoviruses from the Middle-East cluster makes it most likely that Iran is the site of origin of the collected sample.

With the identification of this new orthotospovirus species, the family of *Tospoviridae* is still expanding and has currently reached the number of 30 tentative and established species ([2] and, this report). While this new species has only a small (experimental) host range, its natural host species so far is limited to alstroemeria. With the availability of a specific polyclonal antiserum to AYSV, field surveys could help to identify other natural (cultivated and non-cultivated) plant hosts of this virus. Considering that nothing is known on the prevalence and distribution of AYSV in agricultural and horticultural crops and ornamentals these surveys will also indicate the economic importance of this virus.

During this study a combination of conventional and NGS sequencing approaches has helped to elucidate the entire AYSV genome sequence. The speed and complete data sets obtained by the NGS approach, combined with lowering costs, favor usage of NGS for the elucidation of viral RNA/DNA genomes. However, during this study and our earlier sequence analysis of chrysantemum stem necrosis virus (CSNV) [51] we have observed that the NGS approach may not always provide the entire 5’ and 3’ –terminal sequences and sufficient reads to span the entire A- and U-rich intergenic region. Assembly of the latter reads into one contig is also hampered by the fact that they are relatively short and subsequently fail to properly assemble due to sequence stretches rich in A- and U-residues. Verification of the termini and intergenic region thus still needs to be done by conventional RT-PCR / 5’ and 3’ RACE amplification and Sanger sequence analysis of the amplicons.

## Electronic supplementary material

Below is the link to the electronic supplementary material.

Table S1. Overview of viruses which have been reported from alstroemeria plants (DOCX 17 kb)

Table S2. Accession numbers of orthotospoviral segments and/or corresponding genes, used in this study (DOCX 18 kb)
