# Supplementary material for: Alstroemeria yellow spot virus (AYSV): a new orthotospovirus species within a growing Eurasian clade
Source: Arch Virol. 2018 Oct 4;164(1):117–26. doi: 10.1007/s00705-018-4027-z (PMC6347659; doi:10.1007/s00705-018-4027-z)
Supplement: Supplementary file 1 — Table S1. Overview of viruses which have been reported from alstroemeria plants (DOCX 17 kb) [file 705_2018_4027_MOESM1_ESM.docx]

| Family / Genus | Species (Acronym) | Country | Reference |
| --- | --- | --- | --- |
|  |  |  |  |
| *Alphaflexiviridae / Potexvirus* | Alstroemeria virus X (AlsVX) | Japan | [32] |
|  |  |  |  |
| *Betafelxiviridae / Carlavirus* | Alstroemeria carla virus (AlCV)  Lily symptomless virus (LSV) | UK  UK | [29]  [29] |
|  |  |  |  |
| *Bromoviridae / Cucumovirus* | Cucumber mosaic virus (CMV) | UK, Japan, India | [29, 32, 33] |
| *Tospoviridae/ Orthotospovirus* | Alstroemeria necrotic streak virus (ANSV)  Impatiens necrotic spot virus (INSV)  Iris yellow spot virus (IYSV)  Tomato spotted wilt virus (TSWV) | Colombia  -  The Netherlands, Japan  Japan | [34]  -  [32]  - |
|  | Tomato yellow ring virus (TYRV) | Iran | [35] |
| *Seciviridae / Nepovirus* | Arabis mosaic virus (ArMV) | - | - |
| *Geminiviridae / Begomovirus* | Pepper huasteco yellow vein virus (PHYVV) | Mexico | [36] |
|  |  |  |  |
| *Potyviridae/ Potyvirus* | Alstroemeria mosaic virus (AlMV)*  Alstroemeria streak virus (AlStV)*  Freesia mosaic virus (FreMV) | UK  USA  Italy | [29, 37]  [38, 39, 40]  [41] |
|  | Ornithogalum mosaic virus (OrMV) | The Netherlands | [42] |
| *Secoviridae / Fabavirus* | Broad bean wilt virus-2 (BBWV-2) | Japan | [32] |
| *Virgaviridae / Tobamovirus* | Youcai mosaic virus (YoMV) | Japan | [32] |
| *Virgaviridae / Tobravirus* | Tobacco rattle virus (TRV) | UK | [29] |
|  |  |  |  |

Table S1. Overview of viruses which have been reported from alstroemeria plants.

* Likely strains of the same virus (source: van der Vlugt and Bouwen, 2002)

Title: Alstroemeria yellow spot virus (AYSV): a new orthotospovirus species within a growing Eurasian clade.

Journal: Archives of Virology

Authors: A. Hassani-Mehraban, A.M. Dullemans, J.Th.J. Verhoeven, J.W. Roenhorst, D. Peters, R.A.A. van der Vlugt, R. Kormelink.

corresponding author: R. Kormelink: [richard.kormelink@wur.nl](mailto:richard.kormelink@wur.nl)
