# Supplementary material for: Alstroemeria yellow spot virus (AYSV): a new orthotospovirus species within a growing Eurasian clade
Source: Arch Virol. 2018 Oct 4;164(1):117–26. doi: 10.1007/s00705-018-4027-z (PMC6347659; doi:10.1007/s00705-018-4027-z)
Supplement: Supplementary file 2 — Table S2. Accession numbers of orthotospoviral segments and/or corresponding genes, used in this study (DOCX 18 kb) [file 705_2018_4027_MOESM2_ESM.docx]

| Virus species (acronym) | S segment | | | M segment | | | L segment |
| --- | --- | --- | --- | --- | --- | --- | --- |
|  | NS_S_ | | N | NS_M_ | G_N_-G_C_ | |  |
| Alstroemeria necrotic streak virus (ANSV) | - | | GQ478668 | - | | | - |
| Alstroemeria yellow spot virus (AYSV) | MF469035 | | | MF469034 | | | MF469033 |
| Bean necrotic mosaic virus (BeNMV) | NC_018071 | | | NC_018072 | | | NC_018070 |
| Calla lily chlorotic spot virus (CCSV) | NC_036609 | | | NC_036608 | | | NC_036607 |
| Capsicum chlorosis virus (CaCV) | NC_008301 | | | NC_008303 | | | NC_008302 |
| Chrysanthemum stem necrosis virus (CSNV) | KM114548 | | | KM114547 | | | NC_027718 |
| Groundnut bud necrosis virus (GBNV) | AY871098 | | | AY871097 | | | KX950791 |
| Groundnut ringspot virus (GRSV) | JN571117 | AF513219 | | AF513219 | | | KY350137 |
| Hippeastrum chlorotic ringspot virus (HCRV) | JX833564 | | | JX833565 | | | HG763861 |
| Impatiens necrotic spot virus (INSV) | NC_003624 | | | NC_003616 | | | NC_003625 |
| Iris yellow spot virus (IYSV) | AF001387 | | | AF214014 | | | NC_029799 |
| Lisianthus necrotic ringspot virus (LNRV) | AB852525 | | | - | | | - |
| Melon severe mosaic virus (MSeMV) | KX698422 | | | NC_033833 | | | NC_033834 |
| Melon yellow spot virus (MYSV) | NC_008300 | | | NC_008307 | | | NC_008306 |
| Mulberry vein banding virus (MVBaV) | NC_026619 | | | NC_026618 | | | NC_026617 |
| Peanut chlorotic fan-spot virus (PCFV) | AF080526 | | | KP146141 | | | KP146140 |
| Peanut yellow spot virus (PYSV) | AF013994 | | | - | | | - |
| Pepper chlorotic spot virus (PCSV) | NC_033772 | | | NC_033773 | | | NC_033774 |
| Pepper necrotic spot virus (PNSV) | HE584762 | | | - | | | - |
| Polygonum ringspot virus (PolRSV) | NC_031131 | | | NC_031132 | | | NC_031133 |
| Soybean vein necrosis virus (SVNV) | HQ728387 | | | HQ728386 | | | HQ728385 |
| Tomato chlorotic spot virus (TCSV) | NC_035484 | | | NC_035482 | | | NC_035483 |
| Tomato necrotic ringspot virus (TNRV) | FJ489600 | | | FJ947152 | | | - |
| Tomato necrotic spot tospovirus (TNSV) | KM355773 | | | KX213532 | | | - |
| Tomato spotted wilt virus (TSWV) | NC_002051 | | | AAF80980 | | NC_002050 | NC_002052 |
| Tomato yellow ring virus (TYRV) | AY686718 | | | JN560177 | | | JN560178 |
| Tomato zonate spot virus (TZSV) | NC_010489 | | | NC_010490 | | | NC_010491 |
| Watermelon bud necrosis virus (WBNV) | GU584184 | | | GU584185 | | | GU735408 |
| Watermelon silver mottle virus (WSMoV) | NC_003843 | | | NC_003841 | | | NC_003832 |
| Zucchini lethal chlorosis virus (ZLCV) | KU681011 | | | KU681012 | | | KU681010 |

Table S2. Accession numbers of orthotospoviral segments and/or corresponding genes, used in this study.

Title: Alstroemeria yellow spot virus (AYSV): a new orthotospovirus species within a growing Eurasian clade.

Journal: Archives of Virology

Authors: A. Hassani-Mehraban, A.M. Dullemans, J.Th.J. Verhoeven, J.W. Roenhorst, D. Peters,

R.A.A. van der Vlugt, R. Kormelink.

corresponding author: R. Kormelink: [richard.kormelink@wur.nl](mailto:richard.kormelink@wur.nl)
